# Extensive culturing experiment on *Turborotalita quinqueloba* (spinose planktonic foraminifera)

**DOI:** 10.1093/plankt/fbag064

**Published:** 2026-08-21

**Authors:** Alessio Fabbrini, Julie Meilland, Adele Westgård, Freya E Sykes, Mohamed M Ezat, Audrey Morley

**Affiliations:** Department of Geography, University of Galway, Distillery Road, Galway H91 CF50, Ireland; Department of Earth Sciences, University College of London, Gower Street, London WC1E 6BT, UK; Aix-Marseille Université, CNRS, IRD, INRAE, CEREGE, Technopole Environnement Arbois-Méditerranée BP 80 13545 Aix-en-Provence, Cedex 04, France; iC3: Centre for ice, Cryosphere, Carbon and Climate, Department of Geosciences, UiT - The Arctic University of Norway, Dramsvegen 2019010, 9037 Tromsø, Norway; iC3: Centre for ice, Cryosphere, Carbon and Climate, Department of Geosciences, UiT - The Arctic University of Norway, Dramsvegen 2019010, 9037 Tromsø, Norway; iC3: Centre for ice, Cryosphere, Carbon and Climate, Department of Geosciences, UiT - The Arctic University of Norway, Dramsvegen 2019010, 9037 Tromsø, Norway; Department of Geography, University of Galway, Distillery Road, Galway H91 CF50, Ireland

**Keywords:** Arctic, culturing, foraminifera, plankton, cytoplasm, quinqueloba

## Abstract

Planktonic foraminifera are a ubiquitous group of calcifying marine protists and among the most affected by modern ecological changes due to climate change. Specifically, at high latitudes in the Atlantic Ocean, they are forced to adapt to rapidly warming sea surface temperatures, referred to as the “Atlantification” of the Arctic. *Turborotalita quinqueloba*, a spinose species of planktonic foraminifera*,* is currently proliferating at higher latitudes towards the Arctic Ocean. We conducted a large-scale culturing experiment on *Turborotalita quinqueloba* for 30 days at i UiT-The Arctic University of Norway in Tromsø to investigate its life cycle, reproductive strategies, and how this species reacts to prolonged lab culturing conditions. A total of 357 living individuals were collected and placed in culture using vertical tows deployed to a depth of 50 m at 71° N, 11° E in the Norwegian Sea. Here, we present our live culturing observations and findings, such as the observation of two distinct forms of external structures, and we provide insights for future large-scale culturing experiments on *Turborotalita quinqueloba*.

## INTRODUCTION

Planktonic foraminifera are marine protists widely used in palaeoceanography and biostratigraphy studies (e.g. Banner and Blow, 1965; Blow, 1969; Frerichs, 1971; Cita *et al.*, 1977; Spezzaferri and Spiegler, 2005; Wade *et al.,* 2011; Pearson 2012; Fabbrini *et al.,* 2019 and 2025; Lirer *et al.*, 2019; Lam and Leckie, 2020; King *et al.*, 2020). They preferentially inhabit open ocean waters playing a crucial role in the global carbon cycle by calcifying calcareous tests in the upper portions of the water column (Schiebel and Hemleben, 2017). The geochemical composition of their tests preserves essential climate signals, making them invaluable paleoclimate proxies (e.g. Lear *et al.,* 2000; Elderfield and Ganssen, 2000; Barker *et al.,* 2003). In addition, the biogeographic distribution and abundance of foraminifera species can be used to reconstruct past environmental change on multiple timescales (e.g. Barker *et al.,* 2003; Kucera *et al.,* 2005; Fabbrini and Foresi, 2020). This complex biogeographic distribution translates into a strong provinciality not only in terms of latitude, but also of water masses, depending on physio-chemical properties (e.g. temperature, salinity and nutrient concentration) of the water and on food availability (Jonkers and Kučera, 2015; Schiebel and Hemleben, 2017).

The concentration of planktonic foraminifera in seawater is highly variable globally, however, in e.g. the north Atlantic Ocean, maximum mean concentrations of 31.3 individual per m^3^ occur in the top 50 m of the water column (e.g. Meilland *et al.,* 2019). Such low concentrations raise the question of how planktonic foraminifera maintain sustainable population levels especially when considering their long evolutionary success. For instance, the family GLOBIGERINIDAE prevailed for the entire Cenozoic (Olsson *et al.,* 1999; Fraass *et al.,* 2015) withstanding mass extinctions and major climatic changes, while generating several speciation events (Pearson *et al.,* 2006; Wade *et al.,* 2018; Fabbrini *et al.,* 2021, 2023; Latas *et al.,* 2023; Pearson *et al.,* 2025). Similarly, other planktonic foraminifera adapted to survive and thrive for millions of years, despite changes in oceanic settings, food availability and genetic bottlenecks. The mechanisms allowing such adaptations are still largely unknown. Particularly, it remains to be determined how microorganisms with low concentrations within the water column can successfully colonize and migrate into new habitats, and maintain thriving populations without facing extinctions. Planktonic foraminifera are not free-moving organisms but rather transported as “particles” within water masses, thus their successful migration and colonization of new habitats is a function of survival within oceanic currents and of rate of successful reproduction. Producing a high number of viable offsprings might be the key to sustain a healthy population within changing environments, and it is therefore possible that the modality and mechanisms of reproduction are a determining factor in their ecological success and resilience (Davis *et al.,* 2020; Takagi *et al.,* 2020; Meilland *et al*., 2023; Westgård *et al.,* 2023).

Before Kimoto and Tsuchiya (2006), Takagi *et al.* (2020), Davis *et al.* (2020) and Meilland *et al*. (2023), the scientific consensus assumed planktonic foraminifera were limited to sexual reproduction (gametogenesis), which is a rare and difficult event based on the successful fusion of sexual gametes (karyogamy). Briefly, successful sexual reproduction requires organisms to release gametes at the same time and depth to maximize the chance of gametes to encounter. In addition, many protists, including benthic foraminifera, alternate sexual and asexual reproduction to renovate their genetic pool, while guaranteeing large populations (Goldstein, 1999). Asexual reproduction in benthic foraminifera ensures higher offspring survival rates and rapid population growth, which are fundamental elements for thriving populations. Unlike benthic foraminifera, planktonic foraminifera were considered to have lost the ability to reproduce asexually when they split from their benthic ancestors, until asexual reproduction was independently observed in three planktonic foraminifera species *Neogloboquadrina pachyderma*, *Globigerinita glutinata* (Davis *et al.,* 2020; Westgård *et al.,* 2023; Meilland *et al.,* 2024) and *Globigerinita uvula* (Takagi *et al.,* 2020). These findings indicated that non-spinose and microperforate planktonic foraminifera share the possibility to reproduce in modes similar to their benthic ancestors (Meilland *et al.,* 2024, 2022). The possibility of asexual reproduction could help to explain the survival of planktonic foraminifera in the water column even at low concentrations, and their ability to rapidly colonize new habitats. Investigating the life cycle of *Turborotalita quinqueloba* might unravel whether this fast-migrating species employs multiple modes of reproduction to colonize Arctic environments in response to Atlantification.

Several papers have updated planktonic foraminifera taxonomy and phylogeny in the past decade (Aze *et al.,* 2011; Spezzaferri *et al.,* 2015; Wade *et al.,* 2018; Poole and Wade, 2019; Fayolle and Wade, 2021; Brummer and Kucera, 2022; Fabbrini *et al.,* 2024; Pearson *et al.,* 2025; Lamyman *et al.*, 2026; Fabbrini and Wade, 2026). However numerous questions still remain concerning portions of their evolution history, inconsistent taxonomy for many Neogene and Quaternary species, and some biological issues, such as a complete understanding of all their reproduction modes and their response to environmental forcings. This study focuses on *T. quinqueloba* which is a morphologically simple taxon, having five chambers in the final whorl, usually small (< 200 μm), but characterized by a large variability in its overall test morphology and in the size of the first chamber, i.e. proloculus (Burke *et al.,* 2020). In both benthic and planktonic foraminifera, the size of the proloculus conceives information on how the individuals were produced (Lister, 1894; Goldstein, 1999; Hottinger, 2000). The implications of sexual and asexual reproduction in terms of morphological variability of the phenotypes and eventual repercussions on the taxonomical classifications are unknown and require further study. Nevertheless, a fundamental step to investigating these questions is acquiring, describing and culturing a high number of individuals from one single living population.

### Foraminifera culturing

Live culturing is one of the most direct tools of observation in biology, occupying a central role in understanding and modelling the life cycle of organisms. However, despite advances in cellular biology (Anderson and Bé, 1976; Spindler *et al.,* 1979; Bé *et al.,* 1981, 1983; Caron *et al.,* 1982; Hemleben *et al.,* 1989), the complete and repeated life sexual cycle (from gametes release, gametes fusion, offspring generation, offsprings growth and offspring reproduction) in laboratory conditions for planktonic species is still under described, justifying the necessity to attempt more culturing observational studies.

From the earliest trials in the laboratory to the most recent experiments (Bé *et al.,* 1977; Hemleben *et al.,* 1989; Spero and Lea, 1996; Mashiotta *et al.,* 1997; Lea *et al.,* 1999; Hönisch *et al*., 2021; Davis *et al.,* 2017; Hori *et al.,* 2018; Greco *et al*., 2023; Dong *et al.,* 2022; Meilland *et al.,* 2024 and 2022; Westgård *et al.,* 2023; Sykes *et al.,* 2024; Armitage *et al.,* 2025), culturing planktonic foraminifera enabled observations on biological activities (e.g. from movements and contraction of cytoplasm and pseudopodia to respiration), dietary preferences, feeding strategies, reproduction and pre-reproductive cues, such as symbiont bleaching and spine loss in spinose species (Adshead, 1967; Anderson and Bé, 1976; Erez *et al.,* 1991). All this data improved our understanding of foraminifera life cycle and calcification of their tests. Most of the published culturing experiments concerning life cycle and observations of gametes release (and their synchronicity) are on spinose foraminifera (Adshead, 1967; Bé *et al.,* 1981; Caron *et al.,* 1987; Spero and Lea, 1996; Mashiotta *et al.,* 1997; Uhle *et al.,* 1997; Bemis *et al.,* 1998; Lea *et al.,* 1999; Russell *et al.,* 2004; Kısakürek *et al.,* 2011; Allen *et al.,* 2012; Kuroyanagi *et al.,* 2013; Davis *et al.,* 2017; Hori *et al.,* 2018; Sykes *et al.,* 2024). These culturing experiments involved, for the majority, tropical and subtropical genera, such as *Orbulina*, *Trilobatus*, *Globigerinella* and *Globigerinoides*, while subpolar spinose species such as *T. quinqueloba* have been overlooked.

Von Langen *et al.* (2005), Davis *et al*. (2020), Meilland *et al*. (2022), (2023), Greco *et al.* (2023), Westgård *et al.* (2023) and Sykes *et al.* (2024) widened the understanding of subpolar taxa ecology, publishing foraminiferal culturing protocols targeted to large-scale experiments on both spinose and non-spinose planktonic foraminifera. Following these recommendations provides a solid and tested framework for future studies. The results (i.e. undescribed biological behaviours and reproduction) observed in Meilland *et al.* (2024) and in Sykes *et al.* (2024) call for action especially on other understudied species, such as *T. quinqueloba* which has been extensively used for palaeoceanographic studies at high latitudes (Bauch, 1994; O’Regan *et al*., 2022).

### Turborotalita quinqueloba

*Turborotalita quinqueloba* (Natland, 1938) is a spinose planktonic foraminifera species that evolved in the late Eocene about 37 million years ago (Pearson and Kucera, 2018), making it the longest ranging taxon among living planktonic foraminifera. Within modern planktonic foraminifera assemblages at the sea floor, this morphospecies is usually rare, and while it has a global distribution, it is more common in temperate and subpolar waters. It mainly occupies the photic zone and requires ice free conditions to thrive at high latitudes (Hemleben *et al.,* 1989; Chaabane *et al.,* 2024). *Turborotalita quinqueloba* is a valuable proxy for palaeoceanographic interpretations due to its abundance during interglacial periods in the Norwegian-Greenland Sea (Bauch, 1994) and the Arctic Ocean during the last interglacial period (Vermassen *et al.,* 2023). It is believed that this species reflects the oceanographic changes that occurred during interglacial periods in the North Atlantic and Arctic Oceans when the influence of the Atlantic Water steadily began to reach the Nordic Sea and Arctic Ocean (O’Regan *et al.,* 2022; Vermassen *et al.,* 2023). Higher abundances of this species have been observed in proximity to the ice-marginal zone (Risebrobakken and Berben, 2018; O’Regan *et al.,* 2019), as far north as the southern Arctic Ocean (Carstens and Wefer, 1992) and north of Svalbard in the Arctic Ocean (Anglada-Ortiz *et al.,* 2021) supporting a northward expansion of *T. quinqueloba*. This taxon has also been used in quantitative (i.e. assemblage counts) and morphometric studies (e.g. Bauch, 1994). According to Bauch (1994), variations in test sizes of *T. quinqueloba* might reflect changes in temperature, salinity and nutrient availability in different water masses. However, the size relationship with temperature, salinity or nutrients has never been tested, neither in other oceanographic settings nor in a culture experiment of *T. quinqueloba*.

This study aims to enhance our understanding of the life cycle of *T. quinqueloba,* an understudied spinose planktonic foraminifera species, through a large-scale and extended live culturing experiment. We present here our findings on ontogeny and behaviour of *T. quinqueloba* in culturing conditions. In addition, we discuss the methodology applied and provide supporting documentation with inverted light microscope images and quantitative data. Our observations include photos of external structures in *T. quinqueloba* and evidence suggestive of gametogenesis.

## METHOD

### Oceanographic setting

**Fig. 1:**
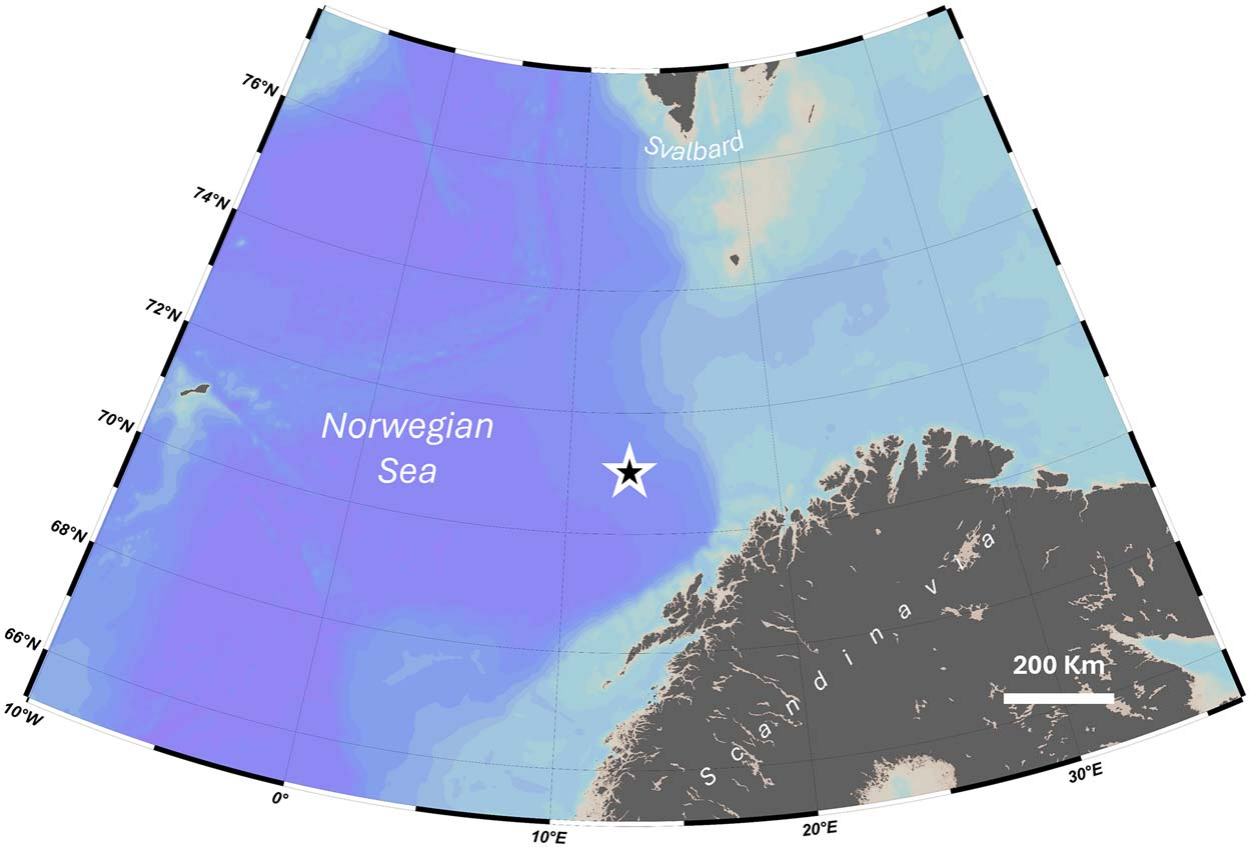
Map illustrating the location of the sampled station for plankton collection (star symbol). Scale bar is 200 km.

Living specimens of *T. quinqueloba* for the culturing experiment were collected from the Norwegian vessel *RV Helmer Hanssen* during the ARCLIM-24-1 oceanographic cruise. The expedition departed from Tromsø (Norway) on the 5 June 2024 and returned to Tromsø on the 11 June 2024. A total of two stations were sampled. The first station (HH24-CTD-847, 70°N and 17°E) was used for seawater sampling only to collect enough seawater needed for foraminifera collection, live culturing on board of the vessel and for the month-long experiment in the laboratory on land. Sea water was collected using 10 L Niskin bottles and filtered using 0.2 μm filters on board of the vessel. The main sampling site used for foraminifera collection was site HH24-NP-866-870 located at 71°N and 11°E (Fig. 1). At this site CTD hydrographic profiles including fluorescence, salinity and temperature were collected down to 300 m water depth. HH24-NP-866-870 is situated at the entrance of the Barents Sea within the Atlantic Water inflow and east of the Arctic Front. Temperature and salinity in the top 50 m ranged between 8.19°C and 6.70°C and between 34.95 and 35.07 PSU, respectively.

### Plankton sampling

Foraminifera were collected using a single net (HydroBios WP2 vertical plankton net) for vertical towing to a maximum depth of 50 m. The net employed had a 63 μm mesh size to maximize collection of early stages of adult planktonic foraminifera from the water column while excluding smaller juveniles. To minimize stress on planktonic foraminifera and to guarantee their best preservation, we applied the sampling protocol described in Westgård *et al.* (2023), Sykes *et al.* (2024) and Meilland *et al.* (2024). Some filtered seawater was poured into 5 L buckets, which served as “waiting rooms” to host the plankton net content before picking. From the buckets the sample was transferred into multiple crystallizing dishes to isolate foraminifera for the final picking. From the crystalizing dishes, foraminifera were picked and transferred into a petri dish prefilled with filtered sea water for a preliminary cleaning bath to remove impurities, other organisms, residues, or to correct possible misidentifications. Then the foraminifera were transferred individually into culturing plates (12 wells each), using a fine picking brush. All culturing wells were prelabelled and prefilled with ~ 7 mL filtered sea water to guarantee optimal conditions as close to the natural environment as possible but cleared from eventual parasites through water filtration and to minimize other biological activity (e.g. phytoplankton blooms). As soon as each culturing plate was filled it was closed with a plastic lid and immediately stored in the cold room on board of the vessel at 8°C temperature, matching the water temperature of the collected plankton’s habitat. The cold room was illuminated with artificial light, guaranteeing 24-hour light conditions matching the environmental summer conditions at the latitude of the sampling station.

A total of 357 healthy specimens were selected and picked abiding by strict criteria based on Meilland *et al.* (2023, 2024) and Westgård *et al.* (2023) to maximize their chance of long-term survival in culture. We picked specimens of *T. quinqueloba* showing the following morphological features: medium size fraction (maximum diameter ~150 μm), five chambers in the final whorl, a thin and transparent test (i.e. not crusted wall texture), evident spines, active rhizopods and dark grey to black cytoplasm within the entire test or in at least four chambers of the final whorl. Whenever extra foraminifera were available and not picked for culturing, due to not satisfying cytoplasm conditions or test size, they were transferred into prefilled plastic culturing bottles and stored in the cold room with other culturing plates to have extra individuals for further investigations.

### Culturing: Feeding and monitoring

After a 24-hour long rest period in the culturing plates, all foraminifera were fed with 20 μL of a solution containing filtered sea water and live diatoms (*Pseudo-Nitzschia* spp.) using a pipette (Greco *et al.,* 2023; Meilland *et al.,* 2024). The diatoms were isolated from the living plankton collected with the foraminifera during the expedition. Diatoms were pipetted out of the seawater contained in the plankton net and transferred into prefilled falcon tubes with filtered seawater and stored to rest and grow in number. After the first feeding, the foraminifera were stored in the cold room on board of the vessel at a constant temperature of 8°C to rest for an additional 24 hours. Upon returning to the Department of Geoscience, UiT—The Arctic University of Norway (on Day 3 since collection), all specimens were relocated into temperature-controlled Friocell EVO incubators programmed to a constant temperature of 8°C for the total duration of the experiment in the laboratory. The incubators are fitted with LED lights shelves enabling a 24-hour light-cycle at an intensity mimicking the conditions of the top 50 m of the water column at the towing latitude in June. Previous ship-based measurements conducted at similar latitudes and season, report values between 30 and 160 photons s^−1^ cm^−2^ over a 24-hour cycle (Westgård *et al.,* 2023), which are also consistent with previous estimates of maximum light intensities of 150 photons s^−1^ cm^−2^ reported for the Nordic Seas (Fram Strait) in June (Manno *et al*., 2012). Accordingly, light conditions in culture were set to a 24-hour cycle split into 3-hour intervals of varying light intensities (e.g. 10–20–30–40–30–20% of 150 photons s^−1^ cm^−2^) as used in Westgård *et al.* (2023). The feeding strategy (frequency and quantity) was constantly reassessed during the culturing experiment, depending on the response of the foraminifera. All 357 individuals were fed six times in total: twice with 20 μL of the diatom solution (Days 1 and 6), followed by four feedings with a 20 μL solution of microalgae (*Nannochloropsis*) on Days 9, 17, 21 and 24. Diatom cultures were maintained and visually assessed prior to feeding to ensure a dense suspension of algal/diatom cells. Because cell concentrations were not quantified during this study, the feeding volume is reported as an operational measure of the feeding protocol rather than a standardized estimate of the amount of food provided. In addition, visual inspection was used as a qualitative assessment of food availability, based on the presence of algal cells in close proximity to the foraminiferal rhizopodial network. The water contained in each culturing well was changed, when necessary, based on water clarity and on the amount of residual organic material visible. The individuals were kept in the described culturing conditions for a total of 29 days in the laboratory at the Department of Geosciences at the UiT-The Arctic University of Norway. All individuals were monitored daily for the total duration of the experiment, using an inverted microscope Zeiss AxioV.A1 and cameras Axiocam 208 colour. On Day 29 all individuals were transferred on numbered and labelled single-well microslides for future analysis and reference.

## RESULTS

### Culturing response

**Fig. 2:**
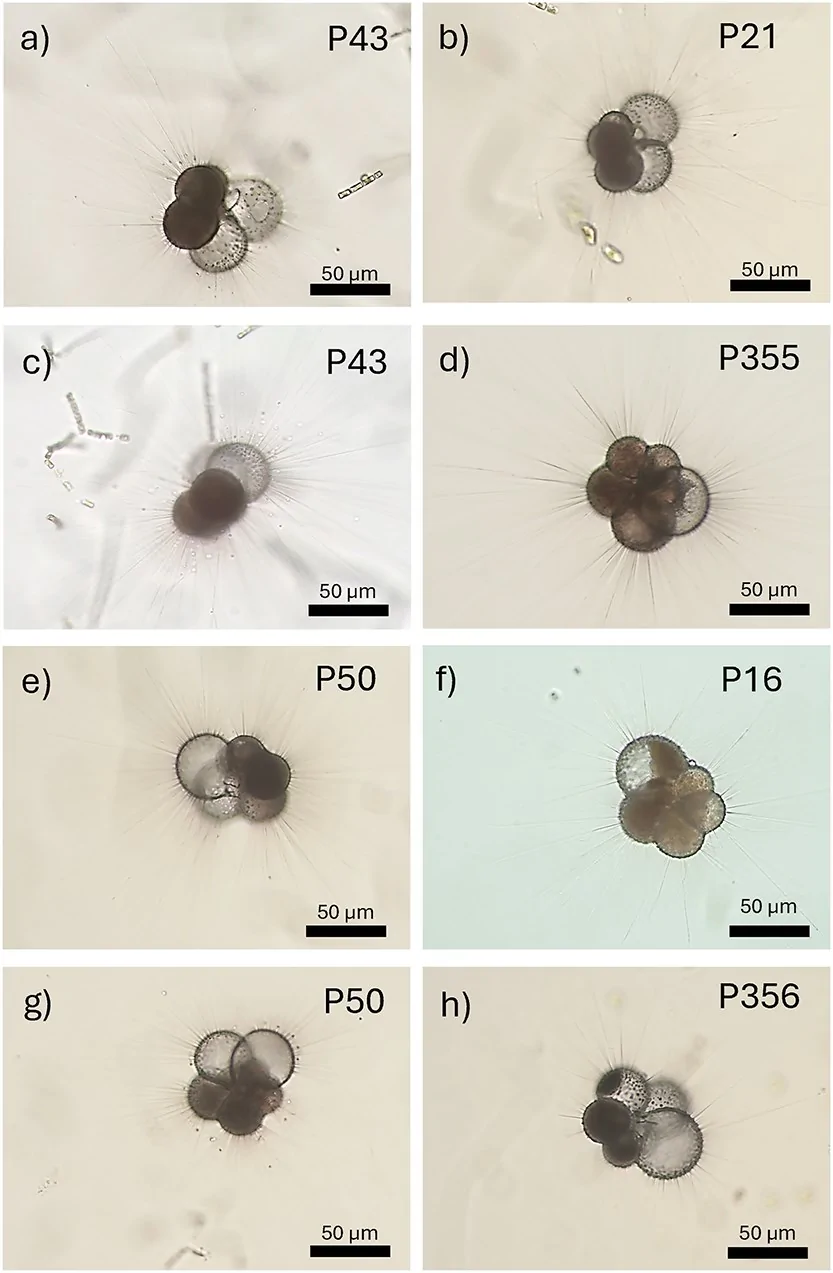
Plate with inverted light microscope photos of selected individuals floating and with spines: a–h. scale bar 50 μm.

During the first day of culturing, two individuals were reported as lost, reducing the total of cultured individuals to 355. The results presented from here on are based on the remaining 355 individuals. After the first feeding with the solution of sea water and diatoms, 300 foraminifera showed signs of recovery and vitality, such as vivid-coloured cytoplasm, extended rhyzopodia and active feeding. The pseudopodia showed motility and signs of a continuous flux of particles between the outside and the inside of the test (Fig. 2). Individuals with extended rhyzopodial networks outside of the test presented dynamic variations of the cytoplasm within the test, thus the cytoplasm did not completely fill the test (Fig. 2a–h; Supplementary Plate-a to d). A total of 18 individuals regrew their spines by Day 4 in culture and started to float on the surface of the culturing well.

Spine regrowth occurred for a total of 87 individuals between Days 4 and 12 in culture, accounting for ~25% of the 355 foraminifera in culture. Thirty-five fully recovered individuals (with spine regrowth) were floating in the culturing wells (corresponding to ~10% of the cultured population), while the remaining 52 remained at the bottom of the culturing wells. A total of 268 individuals did not regrow their spines corresponding to 75% of the total individuals cultured. All individuals which did not regrow spines lived at the bottom of the culturing wells, while continuing all their vital activities (e.g. active feeding and growth). The floating individuals lived on average for a total of 17 days (median is 14 days), spanning from a minimum of 6 days to a maximum of 29 days. The non-floating individuals had a life span ranging from 4 to 28 days.

Two individuals remained floating for the total duration of the experiment (P21 and P50). They adopted a planktonic lifestyle (e.g. floating) on Days 7 and 8, respectively, and survived until the experiment was interrupted. The experiment ended on Day 29 with five foraminifera (P21, P25, P50 and P265) still alive and healthy in terms of cytoplasm colour and active feeding through the rhizopodia.

### Calcification and growth

During the experiment the test of numerous individuals changed their appearance, in terms of thickness of the wall and number of chambers in the final whorl. A total of 295 individuals showed signs of wall thickening by the end of the experiment. Wall thickening was visually and only qualitatively assessed during daily logging, using the inverted microscope. The first individuals to present a thickened wall were registered on Day 4. By Day 12, 205 individuals had a visibly thicker test. After Day 15, wall thickening was observed, on average, in one individual per day.

New or partial chamber growth was documented between Days 3 and 15, and for several individuals the process was extremely slow (>1 day) or aborted. It is difficult to decipher specimens truly calcifying chambers in culture and the ones surviving even when having the final chamber damaged (Supplementary Plate 1-f). Despite the uncertainty on the calcification of the final chamber, five individuals (P89, P149, P150, P299 and P309) were observed to grow an extra aberrant chamber in culture, presenting six chambers in the final whorl, rather than having five chambers as usually occurring in *T. quinqueloba* (Supplementary Plate 2-a and b). These anomalous specimens accounted for 1.4% of the population studied. All foraminfera had five chambers at the moment of collection and picking, thus the sixth chamber of these individuals were added while growing in culture.

### Mortality

**Fig. 3:**
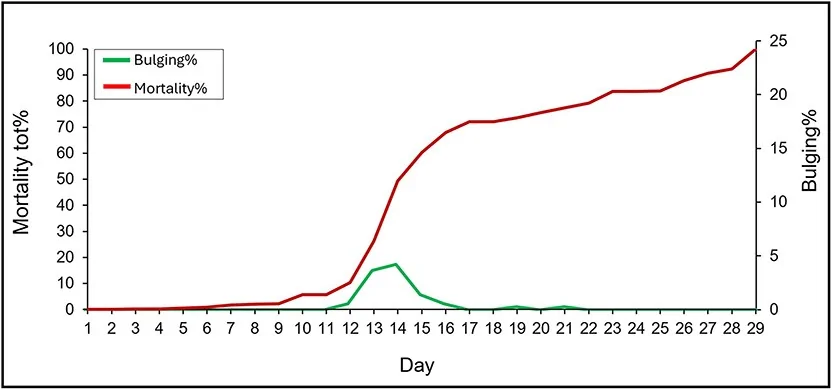
Abundance plots of calculated parameters percentage of the cumulative total mortality (upper line) and apertural bulging percentage (lower line).

Most individuals perished following a steady decline over a prolonged period characterized by paler cytoplasm and absence of pseudopodial activity. Individuals were considered dead after one full day without rhizopodial activity and light-coloured cytoplasm. For simplicity, we will describe the number of dead individuals each day as cumulative mortality hereafter. The cumulative mortality describes the sum of the dead individuals per day expressed as the percentage of the total population in culture (see Fig. 3). By Day 11 in culture, mortality was low (<10%). The highest increase in mortality was observed between Days 12 and 16, reaching its maximum on Days 13 and 14 when on a single day the cumulative mortality jumped from ~ 25% to ~ 50% (Fig. 3). By Day 16, ~ 65% of the initial population was dead. The cumulative mortality progressively reached ~ 80% on Day 22, and reached ~ 90% by Day 27. On Day 28 mortality reached 92% with 28 individuals still healthy, presenting evident spines and floating in the culturing wells. On Day 29 the experiment was terminated and thus the cumulative mortality reached artificially 100%. In addition to these observations, several individuals were contaminated by a “parasite” of unknown origin first observed on Day 10. This contamination may have influenced the high rate of mortality after Day 13, partially compromising our experiment. Further and targeted studies will be necessary to characterize the type of parasitic organism (Supplementary Plate 2-g, h).

### External structures

**Fig. 4:**
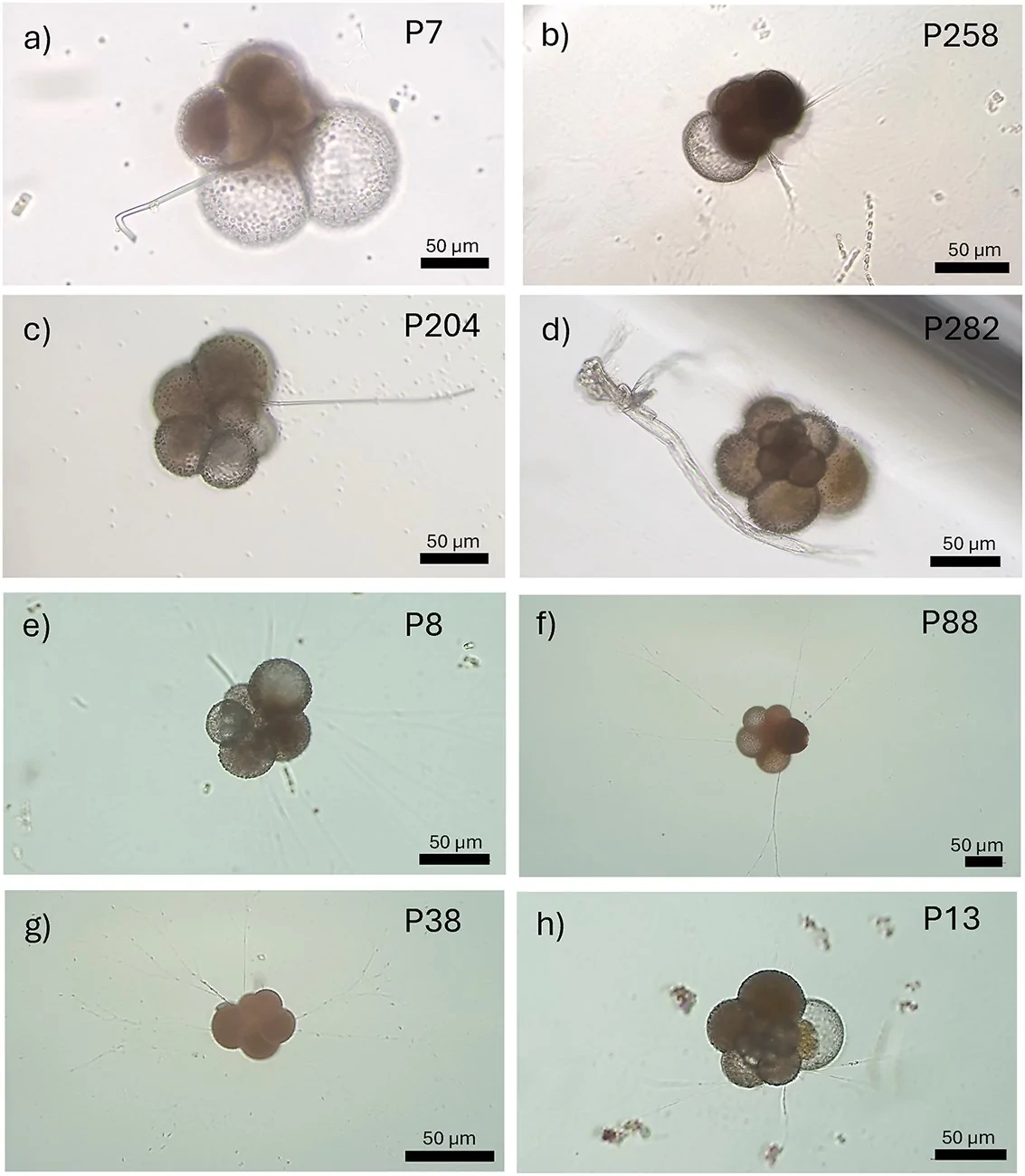
Plate of Axiovert optical images showing external structures and rhizopodial network: a, c and e—Thin; d—Thick; b, f, g and h—Rhizopodial network extroflected. Scale bars are indicated in each image.

Thirty-two individuals presented fibrose extensions from their aperture towards the outside of their tests (Fig. 4 and Table I). Due to the monitoring schedule, only seven specimens with such structures still attached were imaged. Individuals began to develop these features between days 9 and 16, with the highest occurrence on Day 14 with 7 individuals out of 36 presenting an external structure (Table I). We observed two types of structures, a thin type (Fig. 4a, c and e) and a thicker type (Fig. 4d). None of the individuals in this study developed permanent structures, since all were aborted within a few days.

**Table I:** List of external structures with day of first record and imaging.

Individual	Day	Type	Floating	Image (Day no.)
P1	15	Thin	No	No
P3	22	Thin	Yes	No
P7	13	Thin	No	13
P9	20	Thin	No	No
P15	22	Thin	No	No
P76	16	Thick	No	16
P93	16	Thin	No	No
P107	26	Thick	No	No
P119	19	Thin	No	No
P128	22	Thin	No	No
P146	20	Thin	No	No
P150	20	Thin	No	No
P151	22	Thin	No	No
P154	19	Thin	No	No
P169	14	Thin	No	No
P170	14	Thin	No	No
P171	14	Thin	No	No
P172	14	Thin	No	No
P173	14	Thin	No	No
P174	14	Thin	No	No
P198	20	Thin	No	No
P204	13	Thin	No	13
P227	17	Thin	No	No
P248	19	Thin	No	No
P252	14	Thin	No	No
P274	17	Thin	No	No
P281	9	Thin	No	No
P282	9	Thick	Yes	9
P283	26	Thin	No	No
P290	26	Thin	No	No
P306	9	Thin	No	No
P313	15	Thin	No	No

We referred to Greco *et al.* (2023) for the nomenclature of the structures observed. Specimen P7 displayed a thin and short “root,” with a right-angle termination, giving the root an “L-shaped” morphology (Fig. 4a). This root was transparent, 110 μm long and displayed a rounded cross section with a smooth surface. Specimen P204 also displayed a straight, thin root departing from the aperture (Fig. 4c). This thin structure was transparent, smooth, with a rounded cross section, and a total length of 150 μm. Specimen P282 developed the most convolute structure among all the individuals (Fig. 4d). The total length of this structure was > 200 μm and with an average thickness of 8 μm. The thick filament is attached to the test and possibly to the apertural side of the individual. The opposite end of the root has an intricated “knot.”

### Apertural bulging

**Fig. 5:**
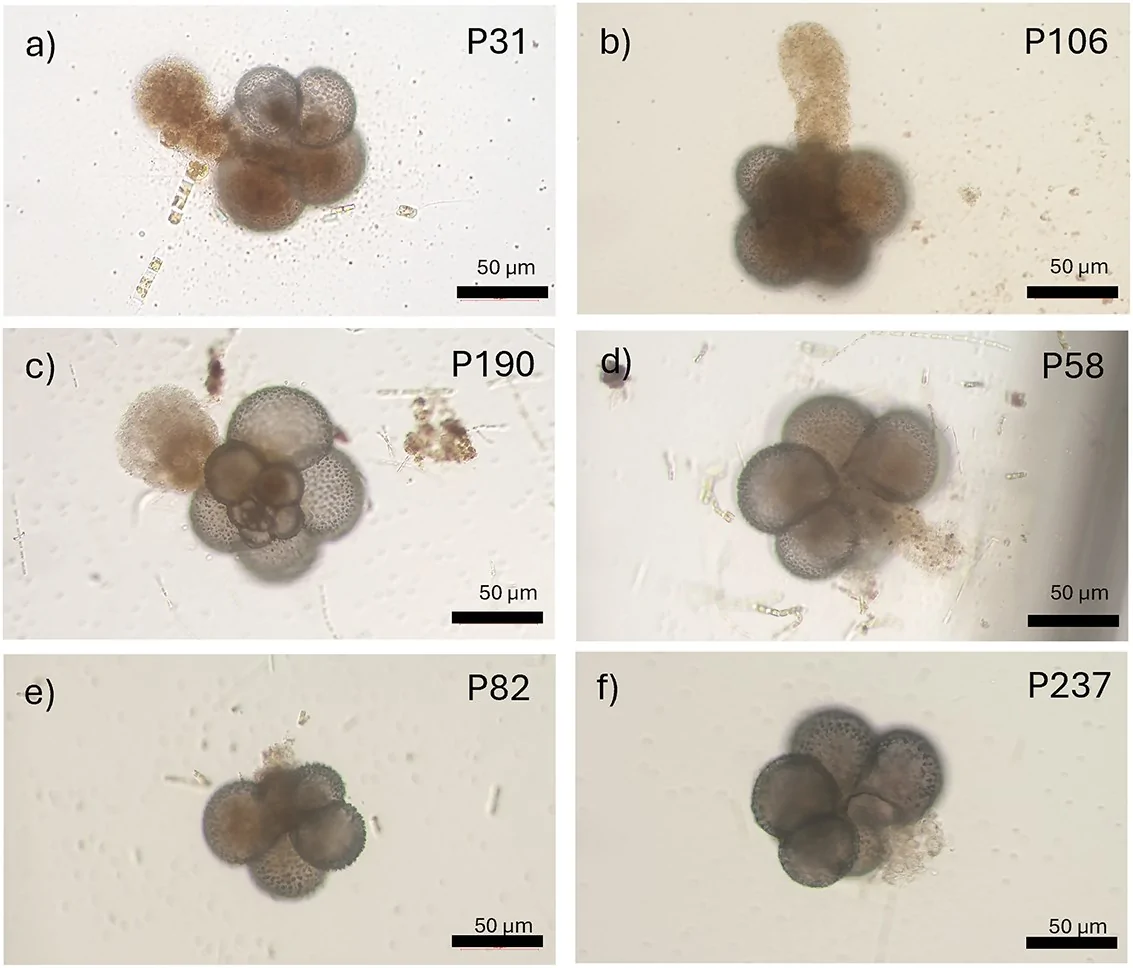
Plate of Axiovert optical images showing examples of different apertural bulges: a–c—“true” bulge (rounded, well-defined and cloudy bulge); d–f—“false” bulge (decaying cytoplasm extruded from the aperture). Scale bar 50 μm.

During the second half of the experiment a series of opaque bulges developed on the aperture of some individuals (Fig. 5). These bulges were observed between Days 12 and 14, with a maximum peak of 15 individuals (i.e. 4% of the total population) on Day 14 as shown in Fig. 3. In total 37 individuals developed apertural bulges during the entire experiment. Two types of bulging on the aperture were observed, one was rounded and well defined (Fig. 5a–c), and a second type with an irregular shape (Fig. 5d–f). Morphologically, these two types of bulges were named as “true” and “false,” respectively. False bulges were interpreted as extroflections of cytoplasm after death of the organisms, and thus not linked to reproductive events (e.g. gametogenesis). Selected examples of different types of apertural bulges are illustrated in Fig. 5. The co-occurrence of a “true” bulge and other factors, such as a thick wall and death of the organism following the formation of the apertural bulge, were noted for further observations concerning the possible reproductive nature of these “true” bulges (see in discussion). Figure 3 shows an existing relationship between the recording of bulges development (both true and false) and the sharp increase in cumulative mortality recorded from Days 12 to 14 (Fig. 3). A complete quantitative separation of true and false bulges would require a more extensive photographic documentation to reassess the number of false bulges, thus Fig. 3 shows the total number of bulges observed during the experiment.

## DISCUSSION

### Potential ecological strategies supporting rapid colonization of the Arctic

This experiment aimed to culture *T. quinqueloba* to study whether its life cycle or reproductive strategies could explain the fast colonization of this taxon in the Arctic in response to the ongoing Atlantification. Observations of asexual reproduction in *T. quinqueloba* would have provided a possible explanation for its northward migration and its ability to colonize the Arctic during favourable conditions like past interglacials (Bauch, 1994; Vermassen *et al.,* 2023; Chaabane *et al*., 2024; Ying *et al.,* 2024). However, in this study we did not observe this mode of reproduction. This lack of observation could be interpreted in multiple ways. First, this species might have a different life cycle than *Neogloquadrina* and *Globigerinita* in which it may exclusively reproduce sexually. It is also a possibility that asexual reproduction occurs in an even lower proportion (~3% of the population for *Neogloboquadrina pachyderma*, Meilland *et al.,* 2023) in spinose species and/or only at a specific time (e.g. immediately after the spring bloom). Alternatively, this could be a byproduct of the culturing conditions, which were potentially not the optimal conditions for asexual reproduction to occur. Finally, the non-observation of asexual reproduction could be the result of the high mortality occurring in the second half of the experiment, which limited the number of healthy individuals surviving until the end of the experiment. Even though we did not observe asexual reproduction here, we believe that future culturing studies might benefit from our results to improve the chance of future success.

Alongside reproductive strategies, the success of a species to colonize a new environment may also be associated with its ability to adapt quickly to different food sources. In this experiment *T. quinqueloba* was fed using living diatoms. However, the species has also been reported to prey on copepods and to be most abundant when fuco-flagellates are dominating the phytoplankton community in the Barents Sea (Meilland *et al*., 2020). These observations suggest that this taxon is adaptable to a variety of feeding strategies; however, the preferred food source that allow *T. quinqueloba* to thrive is still unknown. Meilland *et al*. (2020) suggested that food quality rather than quantity might be a driver for the high abundance of *T. quinqueloba* in the Barents Sea. A recent modelling study also indicated that prey availability and quality is key in thermal acclimatization under a changing climate (Ying *et al.,* 2024). In Volkmann (2000), the distribution of *T. quinqueloba* is inferred to follow the occurrence of other zooplankton, such as Calanoid copepods, which are reported to be the main food source of spinose planktic foraminifera (Spindler *et al*., 1984). Specifically, *T. quinqueloba* is associated with a specific species of copepods (e.g. *Calanus finmarchicus*), suggesting that this species could be its preferred prey. However, if this is the case, food availability cannot drive the rapid colonization of *T. quinqueloba*, since the distribution of Calanoid copepods has diminished at high latitudes in response to Arctic Amplification (Garzke *et al.,* 2015).

To fully explore the possible link between different food sources, reproductive modes and longevity future culturing studies are required. For example, a large-scale experimental design exploring different food sources could test if different modes of reproduction require a different amount of energy provided by a specific food source (e.g. copepod-based vs diatom-based). Such experiments might also clarify which mechanisms, and external factors drive *T. quinqueloba* towards higher latitudes.

In other species asexual reproduction has been described as a rare phenomenon, thus a high number of healthy surviving individuals is a prerequisite to observe it (Schiebel and Hemleben, 2017; Meilland *et al.,* 2023). For instance, in the case of *Neogloboquadrina pachyderma,* ~43% of a natural population will reproduce sexually and ~3% will reproduce asexually, while ~54% of the population will die without reproducing (Meilland *et al.,* 2023). High mortality due to external factors, such as parasites, might have additionally prevented the observation of asexual reproduction during this experiment. The data presented here on this regard are thus insufficient to rule out if *T. quinqueloba* undergoes asexual reproduction. Nevertheless, we address in the sections below a series of observations concerning inferred gametogenesis (phase of sexual reproduction) and we discuss newly observed biological structures in *T. quinqueloba*.

### Potential signs of gametogenesis

Gametogenesis is the release of gametes into the surrounding environment during the final phases of sexual reproduction. A series of sequential events anticipates gametes release in planktonic foraminifera, such as a change in cytoplasm colour, spine loss, wall thickening, the potential addition of a kummerform final chamber, formation of an apertural bulge and death of the parental individual after reproduction (Spindler *et al*., 1979; Hemleben, 1979; Bé *et al.,* 1983; Hemleben *et al.,* 1989; Schiebel and Hemleben, 2017). We interpreted the event of gametogenesis only when a combination of such factors co-occurred in the same individual. Thus, the apertural bulges registered were considered gametogenic only when paired with the absence of spines, thick walls and subsequent death of the organism. During this experiment apertural bulges were documented and imaged (Fig. 5), but gametes were not directly observed. During our experiment, we documented two types of apertural bulges. One had a clear rounded shape, interpreted as a “true” bulge and evidence of gametogenesis if associated with crust thickening, loss of spines, change in the cytoplasm colour and an empty test at the following check (death). The second type was interpreted as a “false” bulge and not linked to reproduction, but rather to cytoplasm waste, expelled via the aperture after the death of the organism.

In this experiment ~10% of the cultured *T. quinqueloba* population underwent “inferred” gametogenesis. No direct examples of gametogenesis in *T. quinqueloba* have been described in the literature, thus a direct comparison of our results with previous observations for the species was not possible. However, when compared with other species such as *N. pachyderma*, our findings are comparable with the estimated 5% of individuals that reach sexual maturity (Brummer and Kroon, 1988; Meilland *et al*., 2023). Whether this low percentage is a true representation of sexual reproduction for this species, or whether it is linked to the introduction of the parasite, or due to suboptimal culturing conditions, remains to be determined in future experiments.

A noteworthy observation is that the peak in bulging and mortality in our experiment was registered around culturing Day 14 which coincided with the full moon (Days 15 and 16 of culturing (20 and 21 June 2024). This coincidence might indicate a link between the lunar synodic pace and the life cycle of *T. quinqueloba* as discussed for other planktonic foraminifera (Berger and Soutar, 1967; Spindler *et al.,* 1979; Reiss *et al.,* 1984; Bijma *et al.,* 1990; Schiebel *et al.,* 1997; Jonkers *et al.,* 2013; Meilland *et al*., 2021). In support of a cyclical trigger *T. quinqueloba* has been reported to show cyclical abundances in the North Atlantic, suggesting a monthly reproductive cycle possibly triggered by the lunar synodic cycle (Volkmann, 2000). However, more data and observations will be necessary to validate this link.

### Significance of external structures

Ectoplasmic structures are described as either permanent or non-permanent protrusions of cytoplasm documented in both benthic and planktonic foraminifera and other protists (Hottinger, 2000). Ectoplasmic roots were observed first in deep-sea benthic foraminifera by Wollenburg *et al.* (2021) who theorized that they might provide a framework to capture food particles. However, the cases documented among planktonic foraminifera in the scientific literature are far more limited than for benthic foraminifera (Anderson and Bé, 1976; Greco *et al.,* 2023; Sykes *et al.,* 2024). Specifically, three hypotheses have been proposed for the presence of ectoplasmic structures in planktonic foraminifera. Sykes *et al.* (2024) proposed that long ectoplasmisc structures might help planktonic foraminifera to retain their vertical position in the water column similar to the presence of spines that are associated with decreased sinking speeds relative to non-spinose species (Takahashi and Be, 1984). Alternatively, these structures may act like preying tools to maximize feeding encounters and thereby survival of the foraminifera in culture (Greco *et al.,* 2023; Sykes *et al.,* 2024). Shorter roots might help foraminifera in maintaining their position when adopting a benthic lifestyle at the bottom of the culturing well (e.g. Greco *et al.,* 2023).

It is also possible that some of the observed fibrose structures in this and previous studies might have an artificial origin and represent fragments of microplastics or other anthropogenic materials. Potential sources include degradation of plastic culture ware or airborne contamination. However, the repeated observation of similar structures across independent studies, sampling campaigns, and species suggests that they are not solely attributable to artefacts (Greco *et al.,* 2023; Sykes *et al.,* 2024). In particular, comparable features have been reported in multiple taxa, including benthic representatives of the order ROTALIIDA (e.g. Wollenburg *et al.,* 2021), supporting the need for further investigation into their origin and potential biological significance.

Here we assume that the structures observed in this study are similar to the ones reported in Greco *et al.* (2023) and Sykes *et al.* (2024) and do not qualify as microplastic. To exclude the potential of abiotic material we followed Lusher *et al.* (2020) guide for the visual identification of microplastics. Briefly, microplastics can be classified by their shape (beads, fragments and fibres), colour (usually homogenous and brighter than biological material), and size. Following this guide the features reported in this study and in previous published literature (Greco *et al.,* 2023; Sykes *et al.,* 2024), do not fulfil the criteria of microplastics. They do not possess anomalous colours or pigmentations; they are not homogenous in morphology and tend to develop angles and convolute thick frames. Moreover, in Greco *et al.* (2023), the ectoplasmic structures have been described (and published) as freely moving, not attached to the jar wall and were observed in association with food particles on their surface [Supplementary video 1 in Greco *et al.* (2023) available on figshare (10.6084/m9.figshare.22665031)]. Analytical approaches such as micro-FTIR spectroscopy could help determine whether these structures are of biotic or abiotic origin; however, such analyses were beyond the scope of the present study. To acknowledge this uncertainty, we adopt a more descriptive terminology for our observations and refer to “external structures” from here on forward.

Thirty-two individuals developed non-permanent external structures. Specifically, we observed two types of roots. The first and more common was thin and short associated with non-floating individuals (Fig. 4a, c and e). This type of structure appears to be comparable with the thin ectoplasmic root described in Wollenburg *et al.* (2021) in the benthic foraminifer species *Cibicidoides pachyderma*, with the difference that none of the structures observed in our experiment became permanent but were always torn and aborted at some stage. The second type of structure that we observed was thicker, longer and presented a convoluted morphology (Fig. 4d), associated with floating/planktonic-like individuals (Table I). The first type of structures was observed from Day 9, however, no clear relation emerged in our data between the formation of structures and feeding schedule or the concentration of particles in the culture wells (as in Greco *et al.,* 2023). The lifestyle adopted by the specimens in culture seems correlated with the type of structure developed as shown in Fig. 4. Floating individuals (Fig. 4d) had the thicker type of roots, while specimens without spines and non-floating (Fig. 4a and e) had the thinner type of roots (n = 33). Three individuals (Table I) presented a thick root but only one was floating in the culturing wells. Twenty-nine individuals (Table I) showed a thin root originating from the specimen’s aperture including one floating in the culturing well.

Greco *et al.* (2023) described ectoplasmic roots in the non-spinose species *N. pachyderma* and *N. incompta* and ectoplasmic twig-like structures in the spinose species *Globigerina bulloides. Neogloboquadrina* developed two distinct types of structures. The first was a permanent thin root, while the second was non-permanent filopodia-like projection found in *N. pachyderma*. They reported both permanent and non-permanent structures after 13 days in culture, which is similar in timing to what we recorded in our experiment. Sykes *et al.* (2024) conducted a long culturing experiment on the spinose species *G. bulloides* where they observed another permanent type of ectoplasmic protrusion. *Globigerina bulloides* constructed solid organic structures, made of a mix of cytoplasm and biological matter scavenged from the culture medium during the later stages of their lifecycle (Sykes *et al.,* 2024). These were termed twig-like structures and first appeared on Day 18 of the experiment. The twig-like structures were large, arched and looping, eventually enveloping the entire organism (Sykes *et al.,* 2024), in contrast to the non-permanent structures we observed for *T. quinqueloba*. The structures that we observe in this study seem more comparable with the non-permanent fibrose roots (filopodia-like) reported by Greco *et al.* (2023).

We hypothesize that external structures might be a common feature to all foraminifera. We cannot exclude, though, that it could also represent a culturing artefact. Indeed, our observations on *T. quinqueloba* suggest that the type of structure may be influenced by the lifestyle and/or by the presence of spines (likely influenced by the specimen collection and culture settings). The non-floating individuals presented short filopodia-like protrusion also observed in *N. pachyderma* from Greco *et al.* (2023), while the floating individuals with spines showed thicker and convoluted structures, more akin to the complex twig-like system reported in *Globigerina* by Sykes *et al.* (2024).

## CONCLUSION

This experiment monitored a total of 355 individuals of the spinose species *T. quinqueloba* for one month in culture. We aimed to describe the complete life cycle (from reproduction to the next reproductive event) of a population of this species to answer open questions about their latitudinal shift towards higher latitudes, during the ongoing Atlantification of the Arctic. While the full life cycle, including reproduction, could not be conclusively documented under the present experimental conditions, this study provides new observations on the growth and survival of *T. quinqueloba* in culture. These results help constrain key aspects of its biology and highlight reproduction as a critical target for future experimental work aimed at understanding its ecology and ongoing latitudinal shifts.

In total 87 individuals of *T. quinqueloba* adapted to the culturing conditions with signs of recovery such as coloured cytoplasm, spines regrowth and floating in the culturing well as they would do in the water column. Some other individuals recovered but they did not regrow spines and sunk to the bottom of the culturing wells for the entire duration of the culturing experiment while still remaining alive. By Day 14 in culture, the cumulative mortality reached 50% in conjunction with inferred gametogenic events. Identifying sexual reproductive events (gametogenesis) correctly can be difficult if the gametes and their dispersion are not directly observed, thus we are conscious that our data are partial in this regard. Nevertheless, we maintained the observations gathered as part of our results due to their value in characterizing an understudied species. Furthermore, the total mortality was affected by a parasitic infection after Day 15, preventing a complete analysis of the full life cycle of all 355 individuals. This event inevitably impacted the possibility to observe any potential asexual reproduction, undermining one of the goals of this experiment.

On the other hand, we documented two distinct forms of putative external structures in *T. quinqueloba*: a thin and a thick type. It remains unclear whether such structures occur in the natural environment of the water column or whether they are a byproduct of culturing conditions.

Our observations highlight how *T. quinqueloba* is able to adapt to culturing conditions, and how some undescribed components of its life cycle still require further investigation. Our results provide insights to aid future culturing experiments on this species. These findings aim to promote more studies on this taxon to deepen our understanding of the biology and ecology of planktonic foraminifera in high latitudes. Further research is needed to address the long-term implications of rapid Arctic climate change on *T. quinqueloba* as part of the subpolar marine ecosystem.

## Supplementary Material

Supplementary_material_fbag064
