# Supplementary material for: Extensive culturing experiment on Turborotalita quinqueloba (spinose planktonic foraminifera)
Source: J Plankton Res. 2026 Aug 21;48(5):fbag064. doi: 10.1093/plankt/fbag064 (PMC13496244; doi:10.1093/plankt/fbag064)
Supplement: Supplementary file 1 [file supplementary_material_fbag064.zip › Supplementary_Plate_caption_fbag064.docx]

Supplementary Plate 1 – Plate of Axiovert optical images showing examples of healthy individuals in culture: a-e floating with long spines and rhyzopodia; f-h non floating and with rhizopodial networks extruded out of the test. Note how f) has the final chamber broken but rhizopodia extended and dark cytoplasm. Scale bar 50 µm.

Supplementary Plate 2 – Plate of Axiovert optical images showing examples of aberrant and calcifying individuals while in culture: a-b aberrant specimens with 6^th^ extra chamber added to the last whorl; c-f individual calcifying an extra chamber in culture; g-h example of foraminifera attacked by parasite, note the deteriorating cytoplasm and the rounded unidentified parasite beside the tests. Scale bar 50 µm.
